# Continuous Directional Water Delivery on the 3D-Printed Arrowhead Microstructure Array

**DOI:** 10.3390/ma12071043

**Published:** 2019-03-29

**Authors:** Lihua Liang, Wei Wang, Junjun Chen, Kunpeng Jiang, Yufeng Sheng, Xiang Peng, Aiping Liu, Huaping Wu

**Affiliations:** 1 Key Laboratory of Special Purpose Equipment and Advanced Manufacturing Technology (Zhejiang University of Technology), Ministry of Education & Zhejiang Province, Hangzhou 310014, China; lianglihua@zjut.edu.cn (L.L.); wangweizjut94@163.com (W.W.); junjunchen93_zjut@163.com (J.C.); jkp0130@163.com (K.J.); yufengshengjie@sina.com (Y.S.); pengxiang@zjut.edu.cn (X.P.); 2 Center for Optoelectronics Materials and Devices, Zhejiang Sci-Tech University, Hangzhou 310018, China; liuaiping1979@gmail.com

**Keywords:** unidirectional transport, arrowhead microstructure, superhydrophilic, Laplace pressure, light curing printing

## Abstract

Unidirectional transport is attracting increasing attention in the field of microfluidics, because it does not require an external energy supply. However, most of the current self-driving structures are still plagued with persistent problems that restrict their practical applications. These include low transport velocity, short transport distance, and complex structure. This work reports the design of a new arrowhead microstructure array, on which liquid transport can reach speeds of 23 mm/s and the ratio of transport length to channel width (L/R) can reach up to approximately 40. This structure drives liquid through a unique arrow conformation, which can induce capillary force and arrest the reverse motion of the liquid simultaneously. By means of theory, simulation, and experiment, we have studied the mechanism of liquid transport on this structure. We provide a detailed discussion of the relationship between the velocity of liquid transport and the microstructural dimensions. The findings may inspire the design of novel, unidirectional, liquid-spreading surfaces.

## 1. Introduction

Directional liquid spreading has important ramifications for various organisms, as it can help them survive in harsh environments [1,2,3]. Spider silk can effectively capture water from the morning fog in arid regions via directional droplet motion on its one-dimensional thread-like natural form [4]. With its unique back structure, the fogstand beetle can harvest fog [5,6]. The famous Nepenthes alata plant can induce oriented capillarity, which spontaneously delivers liquid up to the peristome to form a trap in which small insects are caught [7,8,9,10]. These fantastic liquid-transporting processes can inspire the application of current fluid-manipulating systems for labs-on-chips, self-lubrication, oil–water separation, and high-resolution printing [11,12,13,14,15,16].

A unidirectional liquid-spreading system is typically powered by either gradient surface tension, gradient Laplace pressure, or an anisotropic structure [17,18,19,20,21]. In previous studies, gradient surface tension has been generated through manipulation of pH [22], temperature [23], and exposure to UV light [24]. Under this type of driving force, the velocity of liquid spreading can reach about 80 mm/s, but the spreading distance is quite short [25]. Building an anisotropic structure is another way to achieve unidirectional transport of liquids. This type of structure is characterized by complex processing and low-speed liquid transport over short distances [26]. The latter type employs gradient Laplace force, which drives one-way transport of liquids. This is typically observed on spider silk, cactus spines, and Nepenthes alata structures. Systems that employ gradient Laplace force are characterized by high transport speeds and can ensure transport over certain distances [3,6,27]. However, the preparation of gradient Laplace structures is complex and costly. The inadequacies of these structures restrict their application in practice. Therefore, an urgent need exists for new means of liquid transport in the field of microfluidics. 

In this work, we designed an arrowhead microstructure array based on the Nepenthes alata structure. It possesses the same excellent directional liquid transport properties of Nepenthes alata, and the preparation process is much simpler than existing methods. The channel surface is modified to be superhydrophilic, so liquid can easily spread over it. Due to its arrow-like shape, the gradient Laplace force is generated in the transport direction. A retarding force is generated in the opposite direction that hinders backwards transport. The arrowhead microarray exhibits excellent unidirectional liquid transport performance and high transport speed. A Y-type mixer employing an arrow microstructure array displayed a mixing efficiency about five times greater than that of a typical Y-type mixer. We predict these three-dimensional liquid transport surfaces will open up new ways to study unidirectional wetting and will prompt new research and applications.

## 2. Materials and Methods

### 2.1. Theoretical and Simulation Models

#### 2.1.1. Arrowhead Microstructure Array Design

**Figure 1:**
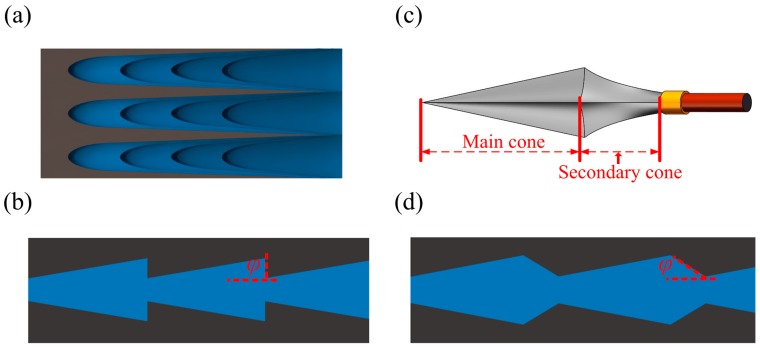
(**a**) The peristome surface of the pitcher plant. (**b**) Schematic diagram of a flat-bottom conical array. (**c**) A typical arrowhead structure that inspired the microstructure design. (**d**) Schematic diagram of arrowhead array.

Through study of the microstructure of the peristome surface in Nepenthes alata [28,29], we constructed a three-dimensional map of its surface. Figure 1a is a simplified depiction of the surface, and the flat-bottom conical structure is shown in Figure 1b. In the flat-bottom conical structure, the cones of each microstructure can generate gradient Laplace forces that drive liquid to move. In the opposite direction of liquid transport, the flat edges of the conical structure provide a blocking force to prevent liquid from moving backwards, which enables autonomous single-direction drive. 

This structure contains a flat-bottom connection in which *φ* + *θ* > 90°, where *φ* is the tilting angle and *θ* is the advancing contact angle. When the liquid passes through this connection, the shape of its surface changes from concave to convex. This produces a pressure gradient that resists further spreading and pins the contact line [30]. When *φ* + *θ* < 90°, the liquid surface remains concave as it passes through the junction and is not met with any extra resistance. Based on this principle, we designed the arrowhead-like microstructure shown in Figure 1c. Arrowheads can be thought of as two conical structures. The main cone is similar to the flat-bottom cone, while the second cone is located at its tail. A simplified model of our arrowhead array is shown in Figure 1d. In this arrowhead structure, the main cone is similar to the flat-bottom conical structure and plays a driving role, while the second cone acts as a buffer. The presence of a transition zone could reduce energy loss during liquid transport, maintain faster transport speed, and allow a longer transport distance.

#### 2.1.2. Theoretical Model of Arrowhead Microstructure Array

**Figure 2:**
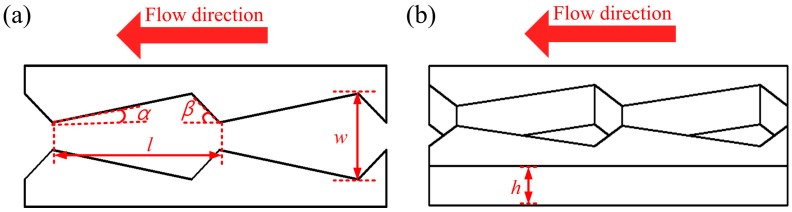
(**a**)The design parameters of the channel. (**b**) The height of the channel.

A systematic investigation of the design parameters of the unidirectional channel could provide additional information for the control of fluid behavior. We therefore studied the mechanism of liquid transport in the arrowhead microstructure. The four following parameters control unidirectional water delivery: the width of the channel (*w*), the tilt angle of the main cone (*α*), the tilt angle of the second cone (*β*), and the length of the microstructure (*l*). These can be seen in the overhead view in Figure 2a, and the height of the channel (*h*) is shown in the side view in Figure 2b.

By studying the relationship between liquid wetting and liquid surface energy, we established the main conical formula of liquid transport in arrowhead arrays [31,32,33]. In the course of liquid spreading, the system potential energy [34,35] is written as

(1) $$U=\gamma_{LV}\cdot \cos\theta \cdot (S_{1}+S_{2})-\gamma_{LV}\cdot S_{3}$$

where (*γ_LV_*) is the surface tension of the liquid/vapor, (*θ*) is the Young’s contact angle, (*S*_1_) is the combined area of the channel walls, (*S*_2_) is the area of the channel bottom, and (*S*_3_) is the area of contact between the liquid surface and air. To calculate the total energy (*U*) in Equation (1), the values of (*S*_1_), (*S*_2_), and (*S*_3_) were first obtained with Equations (2)–(4).

(2) $$S_{1}=\frac{2\cdot x\cdot h}{\cos\alpha }$$

(3) $$S_{2}=\frac{(-2\cdot x\cdot \tan\alpha +2\cdot w)\cdot x}{2}$$

(4) $$S_{3}=S_{2}=\frac{(-2\cdot x\cdot \tan\alpha +2\cdot w)\cdot x}{2}$$

where (*h*) is the height of the channel, (*x*) is the distance of liquid flow, and (*α*) is the tilt angle of the main cone in Equation (2). Because the channel has superhydrophilicity and the viscosity of water is small, and the viscous forces of liquid movement is very small compared with the driving force, we can ignore the effect of viscous forces. According to the Hamiltonian equation of motion, we can get the expression of force by energy.

(5) $$\frac{d}{dt}(p)=-grad(U)$$

After calculating the force, through the relationship between acceleration and velocity, the expression Equation (6) of velocity is finally obtained.

(6) $$\begin{array}{l} v=\sqrt{2\cdot \frac{\gamma_{LV}\cdot \cos\theta \cdot (\frac{2\cdot x\cdot h}{\cos\alpha }-X^{2}\cdot \tan\alpha +w\cdot x)}{(-x\cdot \tan\alpha +w)\cdot x\cdot h\cdot \rho }}\\ +\sqrt{2\cdot \frac{\frac{\gamma_{LV}\cdot x^{2}\cdot \tan\alpha }{2}-\frac{\gamma_{LV}\cdot (-x^{2}\cdot \tan\alpha +2\cdot w\cdot x)}{2}}{(-x\cdot \tan\alpha +w)\cdot x\cdot h\cdot \rho }} \end{array},\;(0\leq x\leq \frac{l\cdot \tan\beta }{\tan\alpha +\tan\beta })$$

When the liquid flows into the second cone, it does not experience capillary force, so it can be viewed as a steady flow of liquid into a ladder-shaped channel. Based on the geometry of the ladder-shaped channel and the initial velocity of the liquid, the change in liquid velocity as it flows through the channel can be determined with Equation (7). (*v*_1_) represents the velocity at which the liquid flows rigidly into the second conical opening.

(7) $$v=\frac{w-2\cdot (\frac{l\cdot \tan\alpha }{\tan\alpha +\tan\beta })\cdot \tan\beta }{x\cdot \tan\beta +w-2\cdot (\frac{l\cdot \tan\alpha }{\tan\alpha +\tan\beta })\cdot \tan\beta }\cdot v_{1},\;(\frac{l\cdot \tan\beta }{\tan\alpha +\tan\beta }\leq x\leq l)$$

Because the flow of liquid proceeds through a series of microarrays, it is not adequate to consider only a single microstructure. The velocity of the liquid in each of the subsequent microstructures must also be determined. As every microstructure is identical, the fluid forces within them are the same. The only difference is the initial velocity of the fluid entering each microstructure. One can simply record the velocity of the liquid at the end of one microstructure and bring it into the next microstructure. The distribution of velocity at each displacement point can thus be known when the liquid flows through the array.

#### 2.1.3. Simulation Model of Arrowhead Microstructure Array

COMSOL Multiphysics is a powerful multiphysical field simulation software for simulation engineering. It helps to simulate various test schemes and reduce test time, especially for high-fidelity simulations of complex geometries [36]. In this paper, the multiphysics coupling feature of “Two-Phase Flow, Level Set” is used to simulate the flow of liquid in the channel. The Level Set interface uses a reinitialized level set method to represent the fluid interface between the air and the water. The model comprised multiple arrowhead microchannels. The entire channel was initially filled with air. The inner walls of the passage were wetted, and the initial velocity and pressure of the liquid were set to zero. Due to the Laplace force and the presence of hydrophilic surfaces, the liquid entered the channel when it came into contact with the opening and could flow freely in the channel. When a full supply of liquid was provided, the liquid continued to moisturize the channel. 

### 2.2. Experiments

#### 2.2.1. Preparation of Arrowhead Microstructure Array

**Figure 3:**
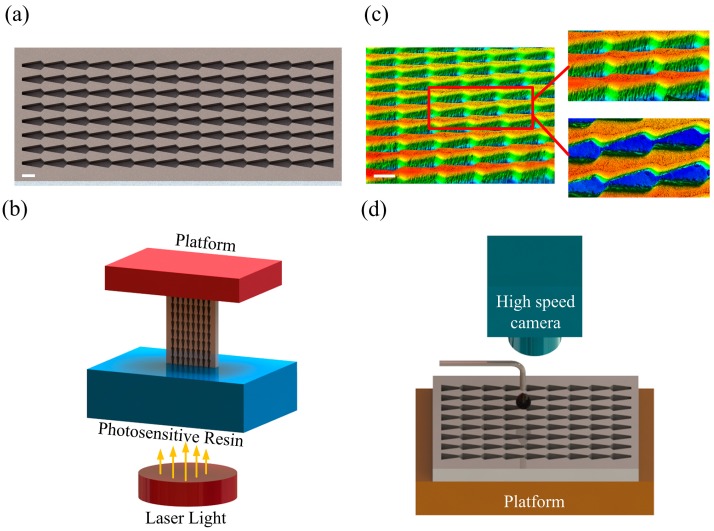
(**a**) Three-dimensional diagram of the arrowhead array (scale bar = 1 mm). (**b**) Conceptual diagram of photocuring 3D printing. (**c**) Microstructural image under a microscope (scale bar = 1 mm). (**d**) Schematic of the high-speed camera.

We employed photocuring 3D printing to prepare our microstructures instead of complicated and expensive photolithography. The ProJet 3510 3D printer used to fabricate the arrowhead array was purchased from 3D Systems (Rock Hill, SC, USA). The advantage of this printer is that it is capable of printing very small objects. The thickness of each layer is 32um and the minimum print precision size is 0.025 mm. It is also easy to operate because it requires only a 3D image of a sample for printing. The 3D model input at the time of printing is shown in Figure 3a, and a conceptual diagram of photocuring 3D printing is shown in Figure 3b. By controlling the projection of UV light into the photopolymer reservoir, each layer of cured resin was printed in accordance with the model. Finally, the finished product was obtained after application of multiple stacked layers.

#### 2.2.2. Structural and Morphological Characterization

Images of the internal microstructure were obtained with a LEXT OLS5000 laser-scanning confocal microscope (Olympus Corp., Tokyo, Japan). Examples are shown in Figure 3c. The total length of a single microstructural channel (l) was 1.8 mm. The length of each main cone was 1.5 mm, the length of the second cone was 0.3 mm, and the height of the microstructure was 0.8 mm. The inclination of the main cone (α) was 10°, while that of the second cone (β) was 41°.

#### 2.2.3. Superhydrophilic Treatment

When the surface of a channel is hydrophobic, it provides no obvious one-way means of liquid transport. The fabricated substrates were treated with oxygen plasma to hydroxylate their surfaces for high surface energies. The specific process was to set the ratio of Ar to O_2_ as 6:1, the flow of gas to 0.1 L/min, and the treatment time to 20 min, and then fluorinate to attain low surface energies. Following treatment, contact angles of less than 10° could be obtained, and superhydrophilicity was possible.

#### 2.2.4. Observation of Transport Phenomenon

For the liquid transport experiments, the arrowhead array was placed on a platform. A high-speed camera was positioned above the array to capture images of the liquid as it was transported. The frame rate of the high-speed camera was set to 600 frames, and the resolution was 640 × 480 pixel. The liquid was fed continuously into the channel through the dropper. The drop height of the liquid was controlled to keep it close to zero, thus removing the influence of gravity, as shown in Figure 3d.

## 3. Results

### 3.1. Comparison of Flat-Bottom and Double Conical Structure

**Figure 4:**
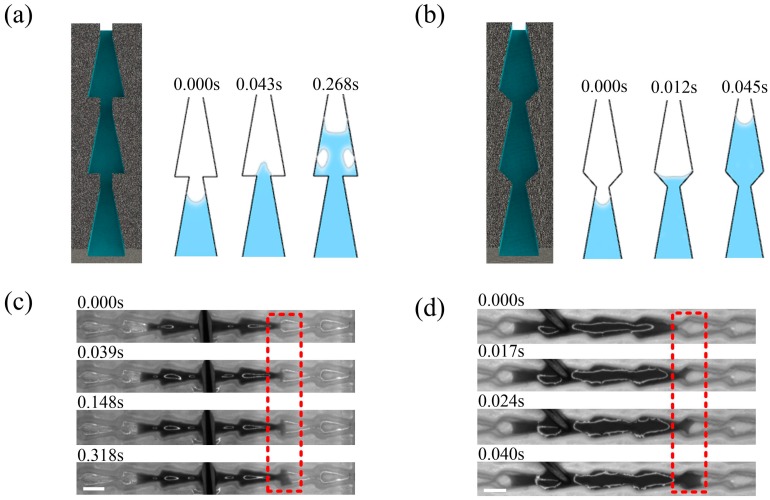
(**a**) Results of the COMSOL simulation with the flat-bottom conical structure. (**b**) Results of the experiment with the flat-bottom conical structure (scale bar = 1 mm). (**c**) Results of the COMSOL simulation with the arrowhead structure. (**d**) Results of the experiment with the arrowhead structure (scale bar = 1 mm).

In the simulation results of COMSOL finite element simulation software, huge resistance to liquid flow occurred at every connection between the microstructures in the flat-bottom conical structure. The blue areas in Figure 4a represent the liquid and the white areas represent air. When the liquid began to break through the narrowest point of the channel and move into the next microstructure, the resistance forced the liquid to form a convex surface, which slowed it down. Even if a large volume of liquid followed, bubbles were produced in the passage due to the blocking effect of the flat-bottom structure, and the gases also had a blocking effect on liquid transport. Additional information about the simulation can be found in Figure S1. This experiment indicated that it took about 0.14 s for the liquid to break through the block in Figure 4b. When the liquid passed through the block with acceleration from the cone in Figure S2, it could reach speeds up to 20 mm/s. The liquid was repeatedly accelerated and decelerated in the channel, and the energy and velocity were dissipated in large quantities.

Results from a simulation with another type of arrowhead structure in Figure 4c showed that it had advantages over the flat conical structure. The trumpet structure allowed the liquid to maintain a concave surface as it passed through the junction. Over the course of transport, the concavity of the liquid surface did not change, which prevented the energy and velocity of the liquid from being lost in transit. The liquid did not produce bubbles as it passed through the junction, which ensured high speed during the transport process. With the high-speed camera, we found that it took only about 0.017 s for the liquid to pass through the junction, which was approximately nine times faster than transport in the flat-bottom conical structure in Figure 4d. This structure not only ensured liquid transport in a single direction, but also prevented the loss of energy during transport. In addition, we compared the two structures with the straight channel structure. It was found that the liquid in the straight channel not only had no unidirectional transport, but also reduced the speed of transport in Figure S3.

### 3.2. Effect of Size on Liquid Transport Velocity

To analyze the relationship between transport velocity and the microstructural dimensions more quantitatively, we also performed theoretical calculations to determine the effects of dimensions on transport. After analysis, we found that the liquid experienced different flow effects depending on the microstructural size combinations.

**Figure 5:**
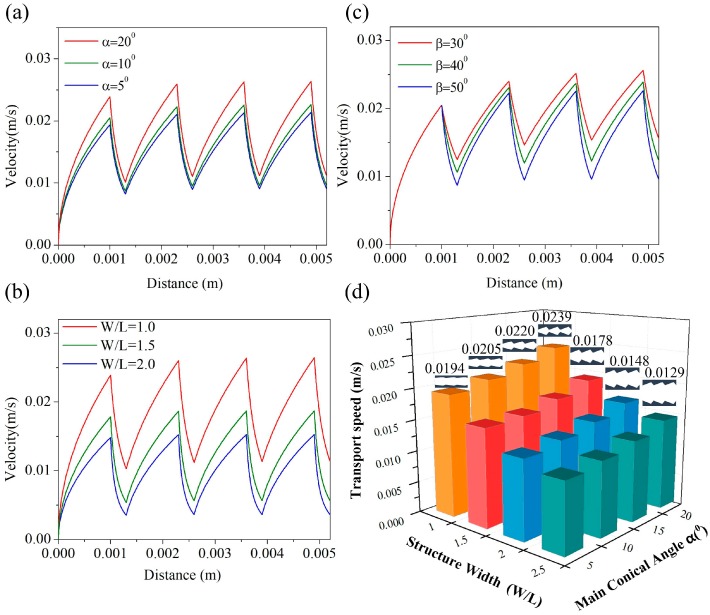
(**a**) The relationship between liquid velocity and angle (α). (**b**) The relationship between liquid velocity and microstructure dimension (w/l). (**c**) The relationship between liquid transport velocity and angle (β). (**d**) Liquid transport velocity with different microstructure sizes.

The more the size of (α) was increased, the faster the liquid was transported. Liquid transport velocity (v) is plotted against displacement (x) for different values of (α) in Figure 5a. The smaller the microstructure size (w/l), the faster the liquid was transported in Figure 5b. We also studied the relationship between the (β) angle in the second cone and transport velocity. It was found that the effects of changes in (β), shown in Figure 5c, were not as significant as those caused by changes in either (α) or (w/l). Among the various dimensions of the arrowhead microstructure, (α) and (w/l) had the greatest effect on the velocity of liquid transport and were the main sources of the gradient Laplace driving force. Here they can be regarded as highly sensitive parameters, while β can be regarded as a low-sensitivity parameter, just as Rezaeiravesh found [37]. It can be seen in Figure 5d that liquid transport was slowest in the microstructure at the largest value of (w/l) and the smallest (α). As (α) was increased and (w/l) became smaller, the liquid transport speed increased, eventually reaching a maximum velocity of 0.0239 mm/s.

### 3.3. Experimental Analysis of Liquid Transport

**Figure 6:**
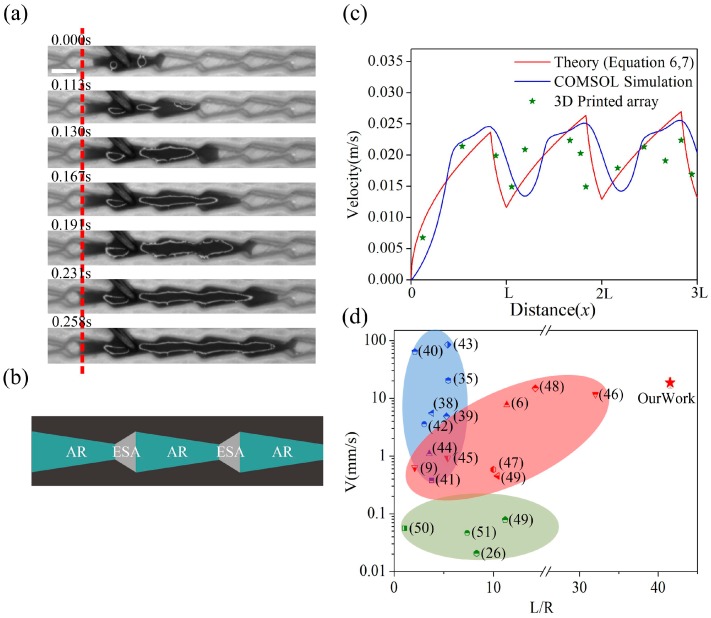
(**a**) Liquid transport process captured with a high-speed camera (scale bar = 1 mm). (**b**) Classification of different regions within the arrowhead microstructure. (**c**) Fitting of theory, simulation, and experimental data. (**d**) A comparison of our work to that available in the literature.

The process by which liquid penetrated across the barriers was recorded with a high-speed camera in Figure 6a. During continuous expansion, the front edge of liquid flow remained concave due to the hydrophilic passage. The liquid moved easily through the narrowest part of the channel to reach the next microstructure. Since the liquid was transported in the arrowhead arrays at high velocity with strong resistance in the opposite direction, very little energy was lost during the transport process. Liquid could also be transported over very long distances, as seen in Figure S4. We divided the arrowhead arrays into several different regions, which are shown schematically in Figure 6b. AR represents the acceleration zone in the arrowhead array and ESA represents the energy storage area in the array. Based on the size of the microstructure and Equations (6) and (7), we determined the relationship between liquid velocity and displacement in the microstructure. According to the results of the COMSOL simulation, we also obtained the corresponding curve in Figure 6c. The simulation results and theoretical predictions plotted in Figure 6c agreed well with the experimental data. With the exception of the initial inflow, the velocity of the liquid increased from zero, then fluctuated within a limited range. The advantages of our structures are illustrated in a comparison between our work and previously published results in Figure 6d. The blue area represents the maximum transport velocity and displacement of the liquid driven by gradient surface tension. The red area represents the maximum transport speed and displacement of the liquid under the gradient Laplacian force. The green area represents the maximum transport velocity and displacement of liquid driven by anisotropic structures. Gradient surface tension drives liquids at high speed over very short distances. The fastest speed was about 80 mm/s, but the distance was only 2–5 [38,39,40,41,42,43,44]; (L) is the length of the liquid flowing in the channel and (R) is the width of the channel. The liquids driven by gradient Laplace force have favorable velocities and transport distances. However, the structures used for these studies are complex, and their preparation is difficult [6,9,45,46,47,48,49]. The average velocity of liquids driven by various anisotropic structures is below 0.1 mm/s, and the transport distance (L/R) is generally less than 20 [26,49,50,51]. Our arrowhead array structures exhibited both high transport speed and long transport distance simultaneously.

**Figure 7:**
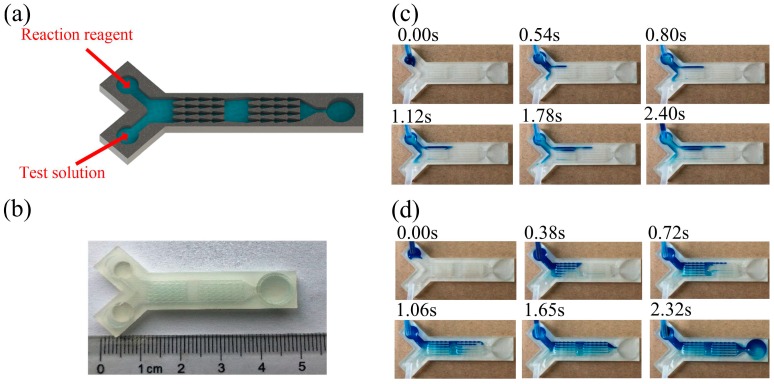
(**a**) Three-dimensional schematic of Y-type mixer. (**b**) The scale of the printed microstructure. (**c**) Mixing results from the Y-type mixer with a straight internal channel. (**d**) Mixing results from the Y-type mixer with an internal arrowhead channel.

The independent unidirectional conveyance capacity of the arrowhead microstructure array enables self-driven fluid transport, and it provides a basis for constructing a pump-free microfluidic channel device. As important components of microfluidic chips, micromixers are used widely for biochemical reactions and analysis. The traditional mixer requires large quantities of reagents and long reaction times. We built a miniature mixer with the arrowhead arrays, which was self-driven and allowed fast flow rates. The detection solution and reaction reagent were introduced at two entry points by continuous flow injection, as shown in Figure 7a. The two liquids were mixed before they entered the arrowhead array. While passing through the arrowhead structure, the solution was divided into four smaller streams for more thorough mixing. Under the acceleration of the microstructure, the liquid flowed forward quickly. After several successive passes through these shuttle arrays, the liquids were completely mixed for the reaction in a reservoir pan. Experimental results showed that the total reaction time in the micromixer was shorter than that in a conventional mixer. With a 3D printing technique, we fabricated Y-type mixers with either straight channels or arrowhead arrays. The Y-type mixer was about 5 cm in length, and the volume of the internal mixing channel was tiny. Only a small amount of liquid was needed to complete the reaction shown in Figure 7b. Over the course of the experiment, we observed that the two streams of liquid flowed to each side of the main passage in the mixer with a straight channel in Figure 7c. As the input increased, it slowly converged towards the middle of the channel. Because the driving force of the liquid in the straight channel was relatively small, and there was no obvious accelerating structure, after 2.4 seconds, the liquid was transported to half of the channel, and some channels were still not wetted. The total cost of transporting liquid from the inlet segment to the tail was 8.62 s. The low speed of transport seriously affected the mixing and reaction times in the system. When the arrowhead arrays in Figure 7d were used as mixers, the two strands of liquid were initially mixed on the input side. The mixture was simultaneously and rapidly transported to the next mixing area via the arrowhead array. Transport of the liquid from the starting point of the passage to the end took only 2.32 s, and the transport efficiency was about four times greater than that of the straight channel. Mixing speed and efficiency were thus much higher in the mixer with the arrowhead array.

## 4. Conclusions

The simplified strategy for unidirectional liquid propagation is based on the unique construction of superhydrophilic surfaces. On this structure, the transport velocity of liquid can reach 23 mm/s and the transport distance (L/R) is approximately 40. With photocuring printing technology, a flow channel can be fabricated with desired properties. With a continuous supply of water, the liquid tends to flow in the direction of decreasing channel spacing. We studied the design parameters and mechanisms of the arrowhead structure in detail and found the main cone of the structure to be the primary cause of the driving force. The secondary cone facilitated transition of the liquid to the next microstructure. The results of the COMSOL simulations and experimental observations also showed that the interaction between capillary force and anisotropy of the liquid was the key to directional liquid transport. The mixing rate in Y-type mixers with arrowhead microarrays was nearly four times higher than that of conventional mixers. The findings of this study provide a facile, scalable, and adaptive strategy for achieving unidirectional water delivery without complex processing technology.

## Supplementary Materials

The following are available online at https://www.mdpi.com/1996-1944/12/7/1043/s1. Figure S1: (a) Simulation Diagram of liquid Transport in conical case with flat bottom. The blue area represents the liquid, the white area represents the air. (b) Simulation Diagram of liquid Transport in arrow array. Figure S2: The detailed process of liquid transport over conical structure with flatbottom. Scale bar = 1 mm. Figure S3: (a) Water spread in straight channel, (b) Water spread in flat-bottom conical structure, (c) Water spread in arrowhead structure. Figure S4: (a) The detailed process of liquid transport over arrowhead microstructures. (b) The furthest distance in which a liquid is transported over the arrowhead microstructure. Scale bar = 1 mm.
