# Supplementary material for: Continuous Directional Water Delivery on the 3D-Printed Arrowhead Microstructure Array
Source: Materials (Basel). 2019 Mar 29;12(7):1043. doi: 10.3390/ma12071043 (PMC6480226; doi:10.3390/ma12071043)
Supplement: Supplementary file 1 [file materials-12-01043-s001.pdf]

materials

Supporting Materials

Continuous Directional Water Delivery on the 3D-Printed Arrowhead Microstructure Array

Lihua Liang [1], Wei Wang [1], Junjun Chen [1], Kunpeng Jiang [1], Yufeng Sheng [1], Xiang Peng [1], Aiping Liu [2] and Huaping Wu [1,*]

[1] Key Laboratory of Special Purpose Equipment and Advanced Manufacturing Technology (Zhejiang University of Technology), Ministry of Education& Zhejiang Province, Hangzhou 310014, China; lianglihua@zjut.edu.cn (L.L.); wangweizjut94@163.com (W.W.); junjunchen93_zjut@163.com (J.C.); jkp0130@163.com (K.J.) yufengshengjie@sina.com (Y.S.); pengxiang@zjut.edu.cn (X.P.);

[2] Center for Optoelectronics Materials and Devices, Zhejiang Sci-Tech University, Hangzhou 310018, China; liuaiping1979@gmail.com

* Correspondence: wuhuaping@gmail.com

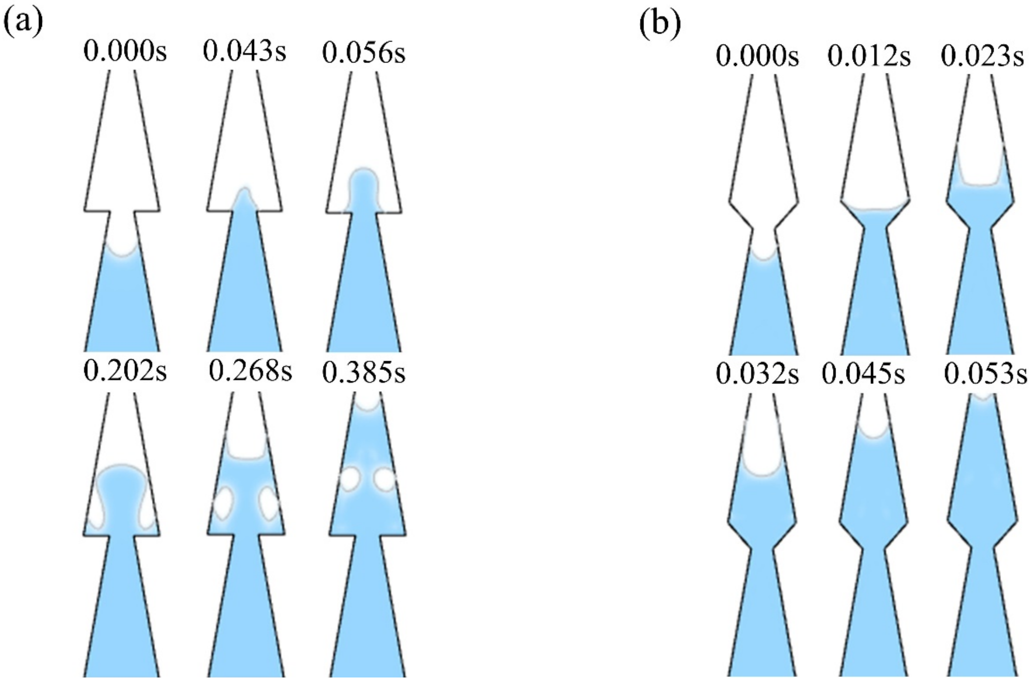

Figure S1. (a) Simulation Diagram of liquid Transport in conical case with flat bottom. The blue area represents the liquid, the white area represents the air. (b) Simulation Diagram of liquid Transport in arrow array.

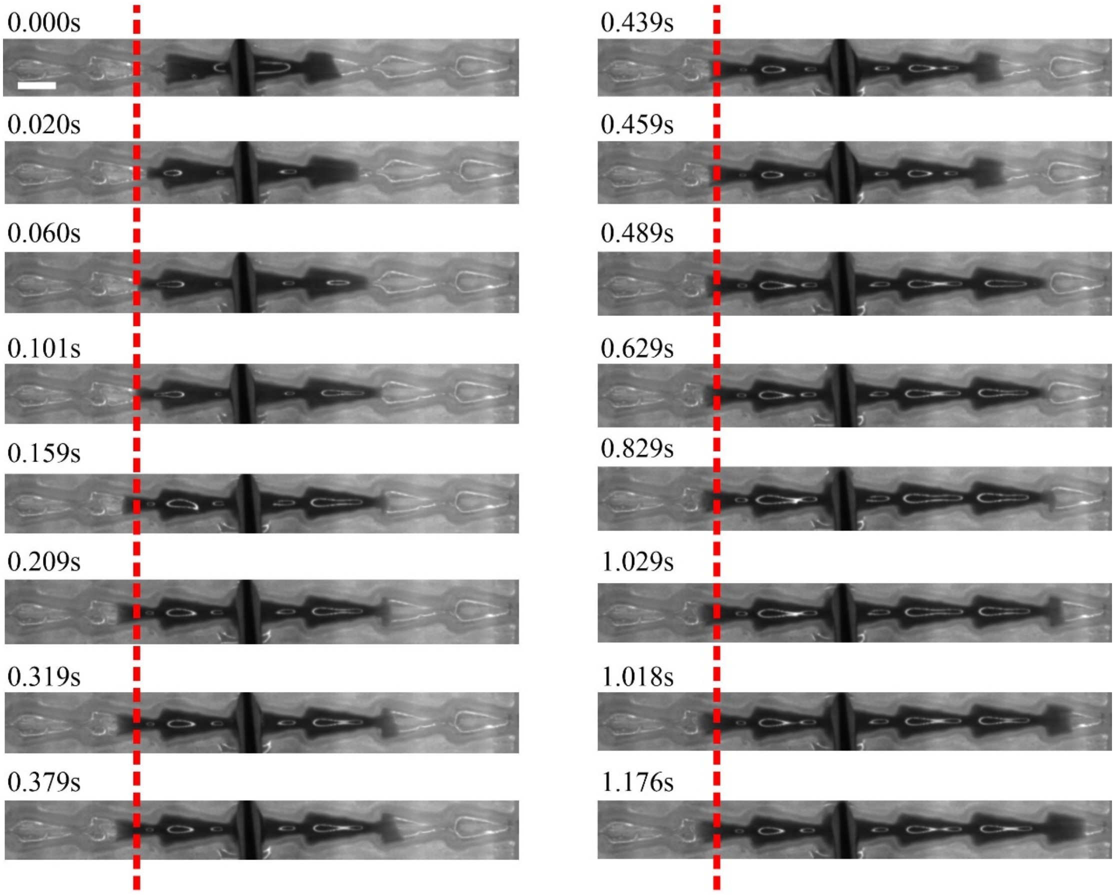

Figure S2. The detailed process of liquid transport over conical structure with flatbottom. Scale bar = 1 mm.

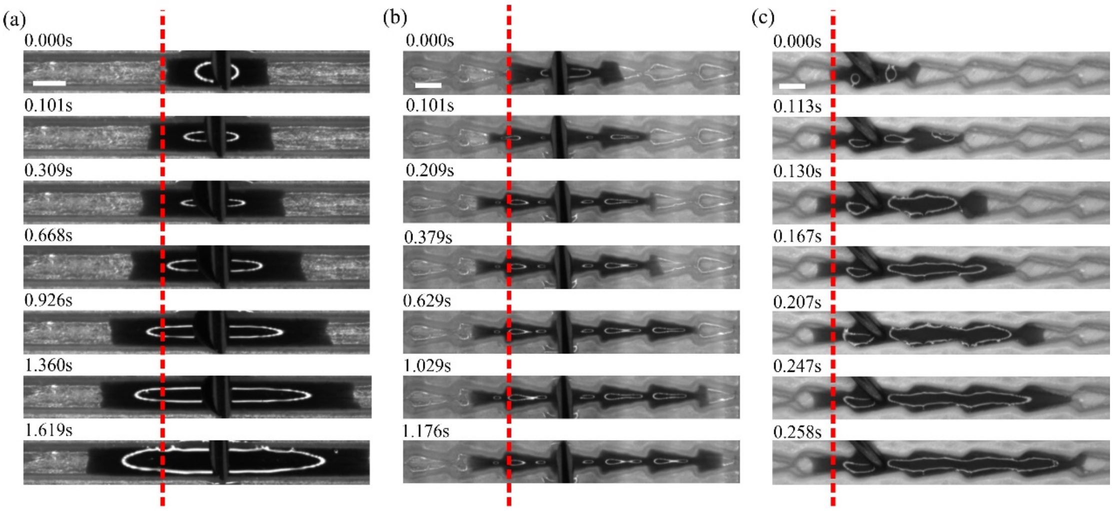

Figure S3. (a) Water spread in straight channel, (b) Water spread in flat-bottom conical structure, (c) Water spread in arrowhead structure.

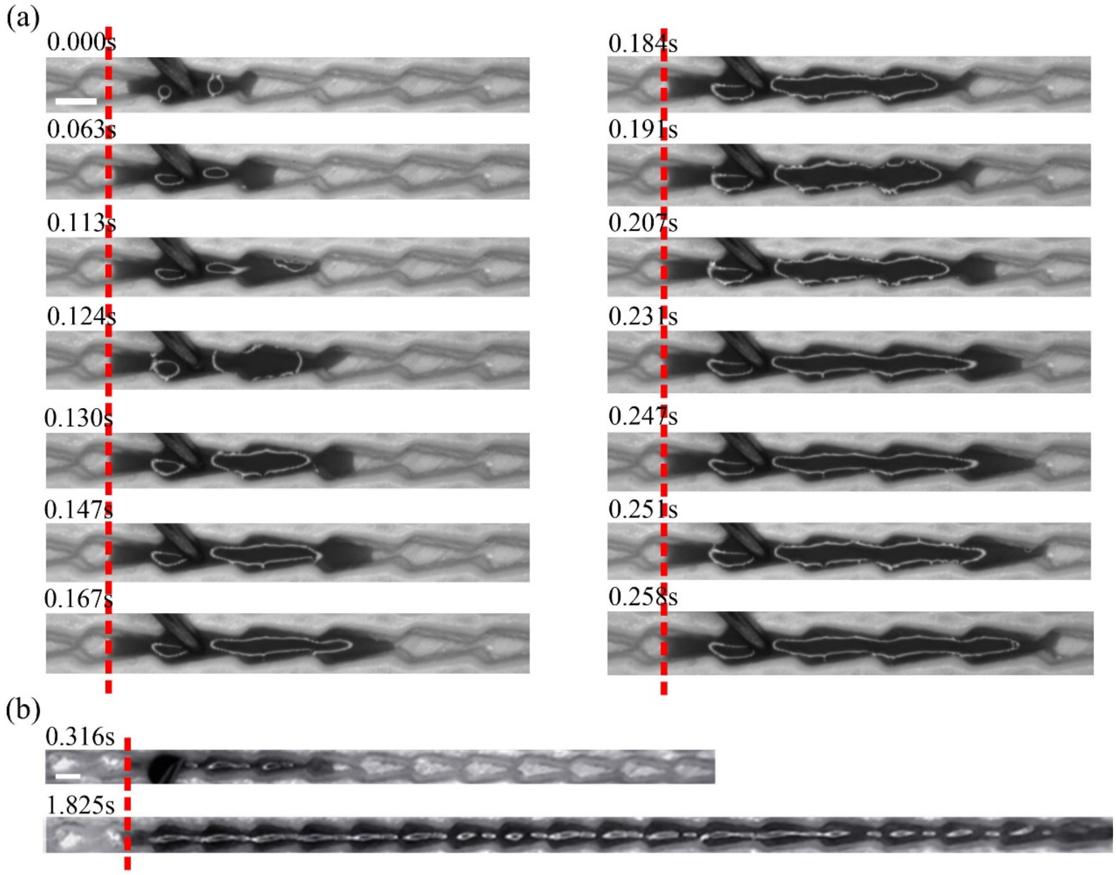

Figure S4. (a) The detailed process of liquid transport over arrowhead microstructures. (b) The furthest distance in which a liquid is transported over the arrowhead microstructure. Scale bar = 1 mm.

© 2019 by the authors. Licensee MDPI, Basel, Switzerland. This article is an open access article distributed under the terms and conditions of the Creative Commons Attribution (CC BY) license (http://creativecommons.org/licenses/by/4.0/).
